# Solitary Apical Lung Mass in a Patient With Cervical Cancer

**DOI:** 10.4021/wjon418w

**Published:** 2012-10-28

**Authors:** Carlos Rodrigo Camara-Lemarroy, Irma Margarita Perez-Rodriguez, Dionicio Angel Galarza-Delgado

**Affiliations:** a Departamento de Medicina Interna, Hospital Universitario “Dr. Jose E. Gonzalez”, Universidad Autonoma de Nuevo Leon. Monterrey, N.L. Mexico. Maderoy Gonzalitos S/N, Monterrey NL. 64700, Mexico

**Keywords:** Cervical cancer, Metastasis, Lung, Adenopathy, Espinocellular

## Abstract

We present the case of a 40 years old female presenting with a solitary apical lung mass, associated Horneŕ syndrome and evidence of medullary compression. Although she had a history of cervical cancer, a primary lung tumor was suspected. Tissue biopsy confirmed cervical cancer metastasis, highlighting the fact that although metastasis usually presents as multiple lung nodules, solitary lesions can be the presenting sign.

## Introduction

Cervical cancer can result in distant metastasis, and recurrence commonly involves pleuropulmonary disease, where the most common presentations are multiple pulmonary nodules and mediastinal and hilar adenopathy [1]. Here we report a case that deviates from this presentation.

## Case Report

**Figure 1:**
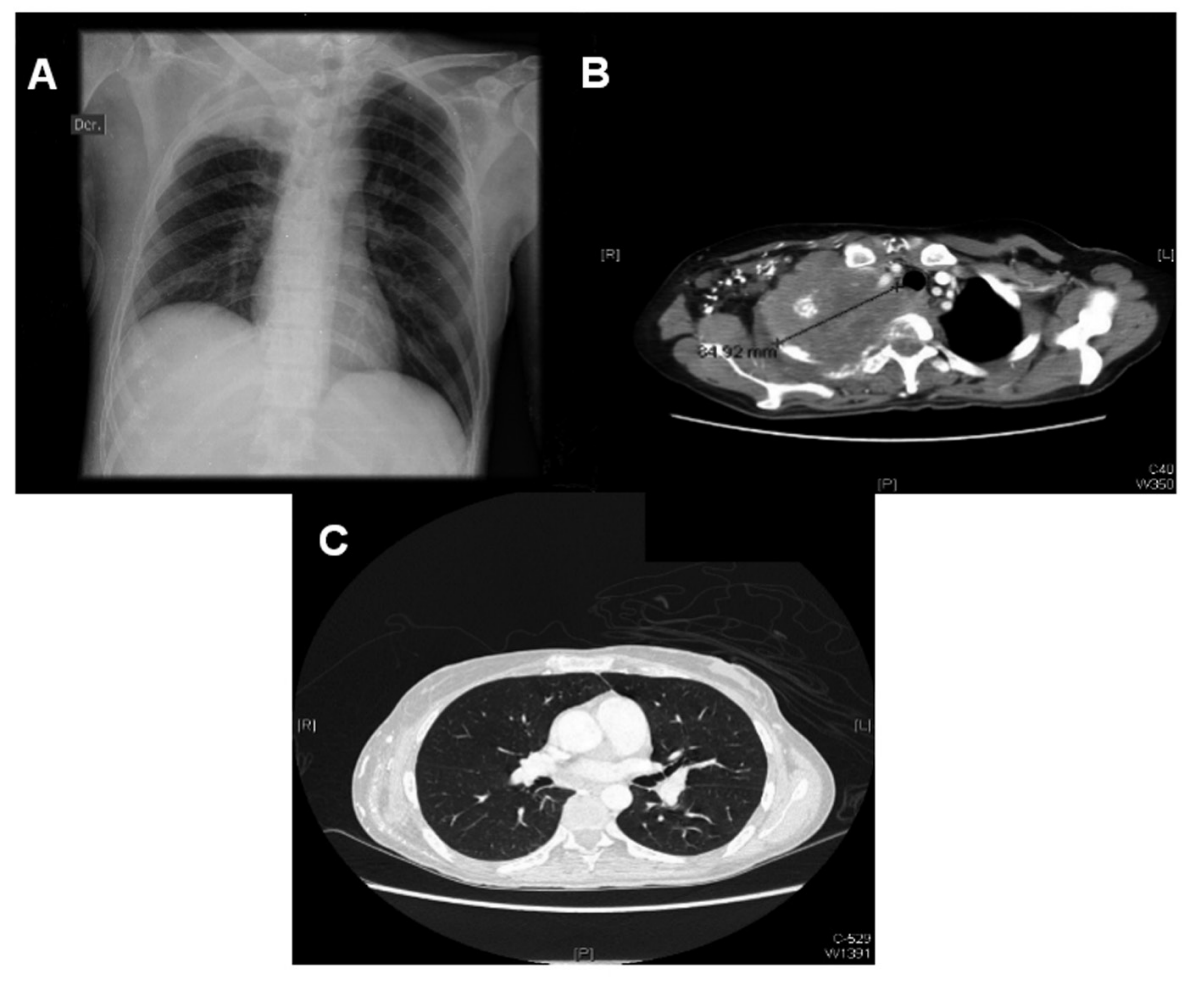
Chest x-ray showing right apical opacity (A). Chest CT showing large apical lung mass, invading vertebral body (B). Rest of lung parenchima is intact, there is no hilar adenopathy (C).

A 40 years old patient with a history of stage IIIb espinocellular cervical cancer diagnosed in 2008 and treated with radiotherapy presented with a history of a right supraclavicular mass that had been growing in the last three months. Four days before her admission, the patient presented sudden paraparesis and bladder incontinence. The neurological exploration also revealed right eye ptosis and a myotic pupil. She also reported weight loss and malaise in the preceding weeks. Her electrolytes, blood chemistries, liver function tests and blood count were normal, except for mild hypoalbuminemia and normocytic anemia. A chest radiograph showed a condensation suggesting a mass in her right lung, without other nodules or widened mediastinum. A chest computer tomography (CT) was ordered, with the finding of a large pulmonary mass invading the thoracic vertebrae (Fig. 1). The patient was immediately started on steroids and local radiotherapy, and a biopsy later showed the mass to be cervical cancer metastasic disease. She had mild clinical improvement.

## Discussion

When cervical cancer metastasis is suspected, CT is the radiographic study of choice [2]. Cervical cancer results in lung metastasis in 32-35% of autopsy findings [3]. The common types of involvement are multiple pulmonary nodules (71%), mediastinal and hilar adenopathy (32%) and pleural metastasis (27%) [4]. Lymphangitic spread, cavitation or endobronchial obstruction are much more uncommon.

The presentation of a solitary apical mass in the right lung, involving medullar compression and Horner’s syndrome, without metastasic disease in other extrapelvic organs or hiliar adenopathy is an unusual presentation of cervical cancer metastasis. Consequently, the differential diagnosis of a second primary lung cancer was considered in this patient before we received her biopsy results. Our patient reinforces the idea that in addition to the typical pattern of multiple bilateral nodules, metastasis may present with solitary nodules, and even normal imaging findings [5].
